# Associations of Physical Fitness and Postural Balance with Psychosocial Well-Being in Early Adolescents: A School-Based Cross-Sectional Study

**DOI:** 10.3390/healthcare14121659

**Published:** 2026-06-11

**Authors:** Juan Aristegui-Mondaca, Gabriel Rodríguez Sepúlveda, Eduardo Guzmán-Muñoz, Jordan Hernandez-Martínez, Joaquín Perez-Carcamo, Edgar Vásquez-Carrasco, Eugenio Merellano-Navarro, Braulio Henrique Magnani Branco, Eduardo Carmine-Peña, Cristian Sandoval-Vásquez, Francisca Peña, Pablo Valdés-Badilla

**Affiliations:** 1 Department of Physical Activity Sciences, Faculty of Education Sciences, Universidad Católica del Maule, Talca 3530000, Chile; juan.aristegui@alu.ucm.cl (J.A.-M.);; 2 Master Program of Physical Activity Sciences, Faculty of Education Sciences, Universidad Católica del Maule, Talca 3530000, Chile; 3 School of Kinesiology, Faculty of Health, Universidad Santo Tomás, Talca 3460000, Chile; 4 School of Physical Education Pedagogy, Faculty of Education, Universidad Autónoma de Chile, Talca 3460000, Chile; 5 Department of Physical Activity Sciences, Universidad de Los Lagos, Osorno 5290000, Chile; jordan.hernandez@ulagos.cl (J.H.-M.);; 6 Department of Education, Faculty of Humanities, Universidad de la Serena, La Serena 1700000, Chile; 7 School of Occupational Therapy, Faculty of Psychology, Universidad de Talca, Talca 3465548, Chile; edgar.vasquez@utalca.cl; 8 Centro de Investigación en Ciencias Cognitivas, Faculty of Psychology, Universidad de Talca, Talca 3465548, Chile; 9 Graduate Program in Health Promotion, Cesumar University (UniCesumar), Maringá 87050-900, Brazil; 10 Carrera de Medicina, Facultad de Medicina, Universidad de La Frontera, Temuco 4811230, Chile; 11 Carrera de Terapia Ocupacional, Facultad de Ciencias de la Salud, Universidad Autónoma de Chile, Temuco 4810101, Chile; cristian.sandoval@ufrontera.cl; 12 Departamento de Medicina Interna, Facultad de Medicina, Universidad de La Frontera, Temuco 4811230, Chile; 13 Sports Coach Career, Faculty of Life Sciences, Universidad Viña del Mar, Viña del Mar 2520000, Chile

**Keywords:** body composition, motivation, muscle strength, proprioception, psychological well-being, self-concept

## Abstract

**Objective:** This study aimed to examine the associations of morphological variables, maximal isometric handgrip strength (MIHS), and static postural balance with self-esteem, motivational climate, school climate, and health-related quality of life (HRQoL) in early adolescents. **Methods:** A cross-sectional study was conducted in 235 Chilean adolescents, in whom morphological variables, MIHS, and static postural balance were assessed using center-of-pressure (CoP) parameters under eyes-open (EO) and eyes-closed (EC) conditions. Psychosocial variables, including self-esteem, motivational climate, school climate, and HRQoL, were evaluated via validated questionnaires. Multiple linear regression analyses were performed to determine associations between physical and psychosocial variables. **Results:** Reduced CoP sway area and lower CoP velocity under eyes-closed conditions were significantly associated with higher self-esteem (R^2^ = 0.168; *p* < 0.001). Greater non-dominant MIHS and younger age were associated with more favorable perceptions of a task-involving motivational climate (R^2^ = 0.438; *p* < 0.001). Higher HRQoL scores were associated with male sex and better postural balance performance. **Conclusions:** Better static postural balance and greater muscle strength were associated with more favorable psychosocial outcomes, particularly self-esteem and HRQoL. However, these findings should be interpreted as associative rather than causal relationships due to the cross-sectional design.

## 1. Introduction

Adolescence, typically ranging from 10 to 19 years of age, is a developmental stage characterized by substantial physiological, cognitive, emotional, and social changes [1,2,3]. During this period, rapid physical growth occurs alongside the development of more complex cognitive abilities, social relationships, and personal autonomy [3]. Consequently, adolescence represents a critical stage for health promotion, particularly regarding the interaction between physical fitness and psychosocial well-being [1].

Globally, about 80% of adolescents do not meet the World Health Organization’s recommendation of at least 60 min of moderate-to-vigorous physical activity each day, making physical inactivity a major public health issue [4,5]. Insufficient physical activity has been linked repeatedly with reduced physical fitness and adverse long-term health outcomes [6]. Psychosocial problems are common in adolescence. Earlier research has reported that about 20–30% of adolescents have low self-esteem, which is closely related to body dissatisfaction and lower psychological well-being [7]. A motivating climate has a strong effect in educational and sports contexts. Task-oriented environments are generally associated with greater satisfaction, persistence, and adaptive psychosocial responses, whereas ego-oriented climates are more often associated with anxiety and disengagement [8]. These results emphasize the necessity of understanding the relationship between physical fitness and psychosocial functioning in adolescence.

Important indicators of healthy adolescent development include body composition, muscle strength, and postural balance [9,10]. These variables reflect not only physical and biomedical health but also psychosocial constructs such as self-esteem, perceived competence, and health-related quality of life (HRQoL) [11]. Previous studies have shown that adolescents with better physical fitness profiles tend to report higher self-esteem, better self-concept, and greater life satisfaction [12]. In this context, muscle strength and postural balance may support psychosocial development by enhancing perceptions of competence, autonomy, and self-efficacy, which are central components of self-determination theory [13]. Postural balance is particularly relevant because sensorimotor control and proprioceptive regulation have been associated with cognitive performance, social participation, and psychological well-being in adolescents [14,15]. Despite this potential importance, postural balance remains relatively underexplored in psychosocial research during adolescence.

Self-esteem is strongly influenced during adolescence by physical attributes such as body composition, muscle strength, and physical appearance, all of which are shaped by peer interactions and social expectations [16]. Previous evidence indicates that greater muscle strength and healthier body composition are associated with more positive body image and higher self-esteem [7,17]. For this reason, self-esteem was selected as the primary psychosocial outcome in the present study. Motivational climate was also considered relevant because it reflects adolescents’ perceptions of achievement goals, competence, and social support within school and sports environments [18]. Task-oriented climates that emphasize effort, cooperation, and personal improvement have consistently been associated with stronger adherence to physical activity and more adaptive psychosocial outcomes [19]. In contrast, environments dominated by peer comparison and performance pressure are more frequently associated with anxiety, frustration, and disengagement [20,21].

HRQoL is a multidimensional construct comprising physical, emotional, social, and academic well-being [22]. Higher levels of physical fitness may be related to better functional independence, better participation in academic work, and more active social engagement [23]. Therefore, HRQoL may be an integrative indicator of how physical functioning is related to the daily well-being and functioning of adolescents [24]. However, most of the extant literature has dealt with physical or psychosocial variables in isolation, limiting conceptual integration and ecological validity. Recent reviews have pointed to the need for more integrative studies of physical and psychosocial dimensions in real-world school settings [25].

From a biopsychosocial perspective, adolescent well-being results from dynamic interactions among physical, psychological, and contextual factors. Within this framework, morphological characteristics, muscle strength, and postural balance may not only reflect physical functioning but may also be associated with perceived competence, self-esteem, motivation, and HRQoL. In particular, self-determination theory and motor competence frameworks suggest that positive perceptions of physical competence and functional ability may contribute to better psychosocial functioning during adolescence [13,26].

Despite the recognized importance of physical fitness and psychosocial well-being during adolescence, important gaps remain in the literature regarding the integrated analysis of morphological variables, objective measures of muscle strength, and static postural balance in relation to psychosocial outcomes among school-based adolescent populations [25,26]. Although body composition and muscle strength have been studied more extensively, less attention has been given to objective indicators of postural control. This gap is important because postural balance may reflect underlying neuromuscular and sensorimotor processes associated with motor competence, perceived physical capability, and psychosocial functioning during adolescence [25,27].

Therefore, the main aim of this study was to examine the associations of morphological parameters, maximal isometric handgrip strength (MIHS), and static postural balance with self-esteem and HRQoL in early adolescents. Secondary exploratory analyses also examined potential associations with motivational climate and school climate variables. These outcomes were considered exploratory because they are influenced by multiple interpersonal and environmental factors and have been less consistently studied in relation to objective physical fitness indicators during adolescence [26]. Based on previous evidence, it was hypothesized that lower body fat percentage, greater MIHS, and better postural balance performance would be associated with higher self-esteem and better HRQoL [17,25,28].

From a theoretical perspective, this study contributes to the growing body of research describing adolescent health as the result of interactions among physical, psychological, and contextual systems. Unlike many previous studies that examined morphological characteristics, muscle strength, or psychosocial variables separately [26], the present study adopted an integrative approach by simultaneously evaluating postural balance, muscle strength, and psychosocial outcomes during early adolescence. By considering physical fitness indicators not only as markers of performance but also as correlates of self-esteem, motivation, and HRQoL, this study aims to contribute to a more comprehensive understanding of adolescent well-being and to inform future longitudinal and intervention-based research.

## 2. Materials and Methods

### 2.1. Study Design

**Figure 1:**
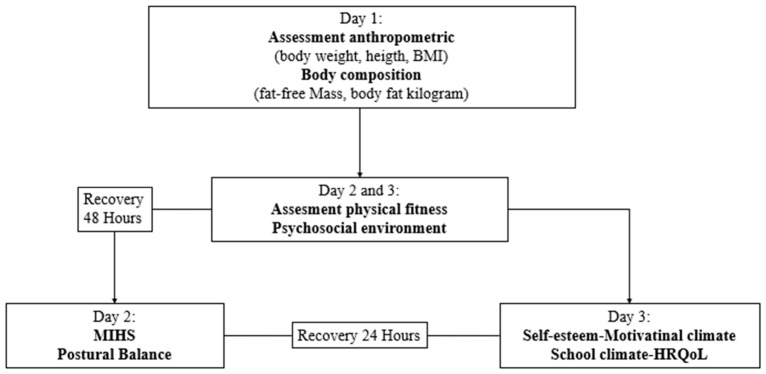
Procedure for measuring body composition, physical fitness and psychosocial environment in adolescents.

This cross-sectional study explored potential associations among morphological, physical, and psychosocial variables in early adolescents within a school-based setting. Due to the observational and cross-sectional design, causal relationships and directionality between variables cannot be established [29]. Participants were early adolescents from Talca, Chile, assessed between April and October 2024. All evaluations were conducted by trained evaluators under standardized laboratory conditions (21–24 °C) during the first academic term. The assessment protocol consisted of three consecutive sessions: Day 1, morphological assessments; Day 2, physical fitness evaluations; and Day 3, psychosocial questionnaires. The sequence of assessments is presented in Figure 1.

### 2.2. Participants

A total of 235 early adolescents participated in the study (mean age = 13.70 ± 1.41 years). According to the World Health Organization, early adolescence includes individuals between 10 and 14 years of age [1]. The final sample consisted of 61 females (26.0%) and 174 males (74.0%). Participants were recruited using a purposive non-probabilistic sampling strategy. Schools were selected based on accessibility, willingness to participate, and logistical feasibility during the assessment period. To reduce potential external interference during data collection, schools with a lower presence of university students completing professional training placements were prioritized. Within the selected schools, all students meeting the inclusion criteria were invited to participate in order to minimize potential selection bias. Therefore, the sample should be considered context-specific and not necessarily representative of the broader adolescent population.

The required sample size was calculated a priori with G*Power software (version 3.1.9.7; Heinrich Heine University, Düsseldorf, Germany) as recommended by Faul et al. [30]. Using a priori power analysis for multiple linear regression with 17 predictors, an alpha level of 0.05, statistical power of 0.80, and small-to-medium effect size (f^2^ = 0.15), we calculated a minimal sample size of 146 people. The expected response rate was 85%; therefore, the sample size had to be increased to 235 people.

In addition, a post hoc power analysis based on the observed effect size of the self-esteem model (f^2^ = 0.20; R^2^ = 0.168) indicated an achieved statistical power of 0.97. This post hoc analysis was conducted for descriptive purposes only.

**Figure 2:**
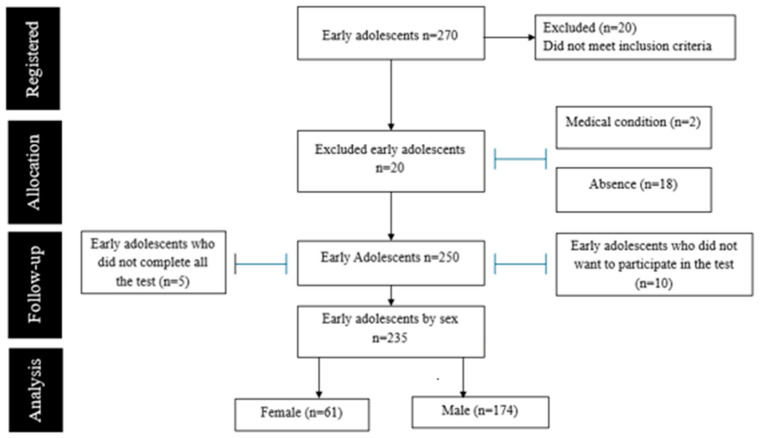
Flowchart of participant recruitment and assessment procedures.

Participants were eligible if they were enrolled in an educational institution and were between 11 and 14 years of age. Exclusion criteria included medical conditions that could interfere with assessment performance and absence on scheduled data collection days. The sample was predominantly composed of male participants, which should be considered when interpreting the findings. The participant recruitment and exclusion process is presented in Figure 2.

Written informed consent was obtained from all participants, together with consent from parents or legal guardians authorizing participation and data use for research purposes. The study protocol was approved by the Scientific Ethics Committee of the Universidad Autónoma de Chile (Approval No. 18-18) and was conducted in accordance with the principles of the Declaration of Helsinki.

### 2.3. Morphological Variables

Standing height was measured using a stadiometer (Seca 220, Gm & Co. Kg, Hamburg, Germany; accuracy: 0.1 cm), and body mass was assessed using a digital scale (Seca 769, Gm & Co. Kg, Hamburg, Germany; accuracy: 0.1 kg). Body mass index (BMI) was calculated as body mass (kg) divided by height squared (m^2^).

Body composition, including fat mass and fat-free mass, was evaluated using an InBody 120 analyzer (Biospace Co., Seoul, South Korea). This device uses tetrapolar bioelectrical impedance analysis with eight tactile electrodes to estimate body composition variables in kilograms.

All anthropometric and body composition assessments were performed according to the guidelines of the International Society for the Advancement of Kinanthropometry (ISAK) [31]. Participants were instructed to avoid vigorous physical activity before testing and to maintain their usual hydration status. Measurements were conducted under standardized school-based conditions and, whenever possible, at similar times of day, following general recommendations for bioelectrical impedance procedures.

### 2.4. Maximal Isometric Handgrip Strength (MIHS)

MIHS was assessed using a hydraulic hand dynamometer (Hand Dynamometer EH101, Camry, Zhongshan, Guangdong, China) following the standardized protocol described by Fess [32]. All assessments were performed by trained evaluators using calibrated equipment and standardized positioning procedures to reduce measurement variability.

Participants were seated with the shoulder slightly abducted, the elbow flexed at 90°, the forearm in a neutral position, and the wrist maintained in neutral alignment. The dynamometer handle was individually adjusted to ensure an appropriate grip position and proper finger flexion with thumb opposition.

Each participant performed three maximal trials with both the dominant and non-dominant hands, with a two-minute rest interval between attempts. The highest value obtained for each hand, expressed in kilograms (kg), was retained for statistical analysis.

### 2.5. Static Postural Balance

Static postural balance was assessed using a force platform (ArtOficio Ltd., Valparaíso, Chile) following the protocol described by Duarte and Freitas [33]. All measurements were performed by trained evaluators under standardized laboratory conditions to improve assessment consistency.

Center-of-pressure (CoP) data were recorded at a sampling frequency of 40 Hz, which was considered sufficient to capture the low-frequency oscillations typically associated with quiet standing postural control in adolescents. Each participant completed 30 s trials under both eyes-open (EO) and eyes-closed (EC) conditions. During testing, participants stood barefoot with their feet positioned at shoulder width and their arms relaxed alongside their body. They were instructed to remain as still as possible throughout the recording period. One standardized trial was performed for each condition.

The CoP variables analyzed mean velocity, anteroposterior velocity (y-axis), mediolateral velocity (x-axis) and sway area. The velocity was calculated as total CoP displacement divided by the recording time and was expressed in meters per second (m/s). This computation gave mean and directional velocities (anteroposterior and mediolateral). Sway area was calculated as the 95% confidence circle around the CoP trajectory and was expressed in mm^2^.

All signal processing and variable calculations were performed using MATLAB R2012a (MathWorks Inc., Natick, MA, USA). Calibrated equipment and standardized procedures were used throughout the assessment process to minimize measurement variability.

### 2.6. Self-Esteem

Self-esteem was assessed using the Rosenberg Self-Esteem Scale [34], a 10-item measure rated on a four-point Likert scale (1 = strongly disagree, 2 = disagree, 3 = agree, 4 = strongly agree). Items 1, 3, 4, 7, and 10 are positively phrased, whereas items 2, 5, 6, 8, and 9 are negatively phrased. The total score ranges from 10 to 40, with values below 25 indicating low self-esteem, 26 to 29 reflecting moderate self-esteem, and 30 to 40 representing high self-esteem. This scale has been validated in Chilean adolescents and has demonstrated acceptable internal consistency (Cronbach’s α = 0.75) [34].

### 2.7. Motivational Climate

The motivational climate in relation to sport participation was measured using the Perceived Motivational Climate in Sport Questionnaire-2 (PMCSQ-2). For the current study, we used a validated Spanish-language version for adolescent populations [35].

The PMCSQ-2 is a 33-item scale with a 5-point Likert scale (1 = strongly disagree to 5 = strongly agree). The questionnaire measures two major dimensions, task-involving climate (Cronbach’s α = 0.87) and ego-involving climate (Cronbach’s α = 0.89), both of which indicate acceptable internal consistency [35].

The task-involving climate dimension consisted of 17 items, with a total score ranging from 17 to 85. The higher the score, the greater the perception of effort, cooperation and personal improvement. This dimension consisted of three subscales: (i) cooperative learning (four items: 11, 21, 31, and 33; score range: 4–20), (ii) effort/improvement (eight items: 1, 8, 14, 16, 20, 25, 28, and 30; score range: 8–40), and (iii) important role (five items: 4, 5, 10, 19, and 32; score range: 5–25) [35].

The ego-involving climate dimension included 16 items, with total scores ranging from 16 to 80. Higher scores reflect stronger perceptions of normative comparison and competitive performance orientation. This dimension was divided into three subscales: (i) punishment for mistakes (six items: 2, 7, 9, 15, 18, and 27; score range: 6–30), (ii) unequal recognition (seven items: 3, 13, 17, 22, 24, 26, and 29; score range: 7–35), and (iii) member rivalry (three items: 6, 12, and 23; score range: 3–15) [35].

### 2.8. School Climate

The version of the School Climate Scale adapted, validated (Cronbach’s α = 0.89) and translated into Spanish by López et al. [36] consists of 18 Likert-type items scored from 1 to 5, where 1 represents strong disagreement and 5 represents strong agreement. The items are grouped into the following subscales: (i) Clear rules and policies (nine items): This dimension explores students’ perceptions of the school’s policies and procedures aimed at reducing school violence. (ii) Teacher support (six items): This dimension assesses the students’ perceived emotional and academic support received from their teachers. (iii) Student participation (three items): This dimension examines students’ feelings about whether they play an important and active role in managing violence within their school.

### 2.9. Health-Related Quality of Life (HRQoL)

HRQoL was assessed using the KIDSCREEN-10 questionnaire, an instrument designed for children and adolescents aged 8 to 18 years. The questionnaire has been validated in Chilean populations and demonstrates high internal consistency (Cronbach’s α = 0.89) [37,38].

The KIDSCREEN-10 consists of 10 items rated on a five-point Likert scale. For items 1 and 9, response options range from 1 = not at all to 5 = extremely. The remaining eight items are scored from 1 = never to 5 = always. Negatively worded items (items 3 and 4) were reverse coded before analysis. Higher total scores indicate better HRQoL [39].

### 2.10. Statistical Analysis

Statistical analyses were conducted utilizing IMB SPSS Statistics version 23.0 (IBM Corp., Armonk, NY, USA). Descriptive statistics, encompassing means and standard deviations, were computed to delineate the sample based on morphological variables, MIHS, static postural balance, and psychosocial measures (self-esteem, motivational climate, school climate, and HRQoL).

Independent-samples *t*-tests were employed to evaluate sex-based disparities. Effect sizes were computed utilizing Cohen’s d and classified as small (0.20–0.49), moderate (0.50–0.79), or large (≥0.80) [40,41]. In the regression analysis, sex was encoded as 0 for female and 1 for male. Chronological age and sex were incorporated as factors in all regression models to somewhat mitigate developmental disparities among subjects. While variables such as age, sex, and body composition were initially included in all models, some were excluded from the final analysis due to their minimal statistical impact in the multivariable models.

Multiple linear regression analyses were performed for each dependent variable: self-esteem, motivational climate (task- and ego-involving dimensions), school climate, and HRQoL. Self-esteem was defined as the primary outcome because of its central role in adolescent psychosocial development and its established relationship with physical fitness and health-related behaviors. Motivational climate dimensions, school climate, and HRQoL were considered secondary exploratory outcomes. For motivational climate analyses, higher scores in the task-involving dimension of the PMCSQ-2 reflected more favorable perceptions of a mastery-oriented and task-focused climate. Regression coefficients were interpreted according to this coding structure.

All models were initially specified using a theory-driven set of predictors, including sex, age, and multiple morphological and physical fitness indicators, to examine their associations with psychosocial outcomes. Multicollinearity was assessed using a variance inflation factor (VIF) and tolerance diagnostics. Because severe multicollinearity was identified between BMI and its components (body mass and height), BMI was excluded from the final regression models to improve model stability and interpretability. In the final models, the highest observed VIF was 8.56 for MIHS, remaining below the predefined threshold of 10 [42]. Complete VIF values for all predictors included in the final models are presented in the Supplementary Materials Table S1).

Model fit was evaluated using adjusted R^2^ values and the corresponding ANOVA *p* values. For each regression coefficient (β), 95% confidence intervals (CIs) were reported. Statistical significance was set at *p* < 0.05 for all analyses. Given the exploratory nature of the study and the multifactorial characteristics of psychosocial outcomes during adolescence, the regression models were intended to identify associative patterns rather than maximize predictive performance or establish causal relationships.

Although multicollinearity diagnostics were acceptable, some CoP variables may reflect partially overlapping biomechanical dimensions of postural control. Nevertheless, these parameters were retained because of their distinct functional and clinical interpretability.

## 3. Results

Descriptive statistics for morphological variables, MIHS, and static postural balance are presented in Table 1. Compared with female participants, male early adolescents were significantly older (*p* < 0.001), heavier (*p* < 0.001), taller (*p* < 0.001), and had greater fat-free mass (*p* = 0.001), as well as higher MIHS values for both the dominant and non-dominant hands (both *p* < 0.001). Additionally, females showed significantly greater mediolateral (ML) velocity under EC conditions than males (*p* = 0.044). These observed differences should be interpreted cautiously, as they may partially reflect maturational differences during early adolescence rather than sex-related differences alone.

Sex-specific descriptive statistics for psychosocial variables are presented in Table 2. Compared with females, males reported significantly higher HRQoL scores (*p* < 0.001). In contrast, females showed significantly higher scores in the task-involving motivational climate dimension (*p* < 0.001), whereas males scored higher in the ego-involving motivational climate dimension (*p* < 0.001). No significant sex differences were observed for school climate.

For the primary outcome, the self-esteem regression model was significant (R^2^ = 0.168, *p* = 0.001). Lower CoP sway area under EC conditions (β = −35.053, *p* = 0.001), lower CoP mean velocity under EC (β = −6.860, *p* < 0.001), and lower CoP mediolateral velocity under EC (β = −5.138, *p* = 0.024) were significantly associated with higher self-esteem scores (Table 3).

Among the secondary exploratory outcomes, the task-involving motivational climate model was significant (R^2^ = 0.438, *p* < 0.001), with younger age (β = −4.485, *p* < 0.001) and greater non-dominant MIHS (β = −0.181, *p* = 0.035) being significantly associated with higher scores (Table 4). In contrast, the regression models for ego-involving motivational climate (R^2^ = 0.008, *p* = 0.346) and school climate (R^2^ = 0.002, *p* = 0.561) were not significant.

For HRQoL, the regression model was also significant (R^2^ = 0.158, *p* < 0.001). Lower CoP mean velocity under EC (β = −57.581, *p* = 0.002), lower CoP sway area under EO (β = −30.003, *p* < 0.001), and male sex (β = 3.592, *p* < 0.001) were significantly associated with higher HRQoL scores (Table 5).

## 4. Discussion

This cross-sectional study investigated the relationships among morphological variables, MIHS, static postural balance, and psychosocial outcomes in early adolescents. The primary findings indicate that improved postural balance performance, especially in eyes-closed settings, correlated with elevated self-esteem and enhanced HRQoL. These findings augment prior studies suggesting a positive correlation between physical competence, motor performance, and psychological well-being in adolescence [7,12,17,26]. Younger age and more non-dominant MIHS were correlated with more positive evaluations of a task-involving motivational climate. Nonetheless, due to the cross-sectional design and the limited explanatory capacity of various regression models, these results should be regarded with caution as associations rather than causal relationships.

This study established a relationship between enhanced static postural balance under EC conditions and increased self-esteem. Adolescents with a reduced CoP sway area, lessened mean CoP velocity, and lower mediolateral velocity exhibited elevated self-esteem levels. The results correspond with previous studies demonstrating that increased physical competence and superior functional profiles are associated with better self-perceptions and psychological adjustment in teenagers [7,17,25]. From a biomechanical perspective, the features of CoP displacement reflect neuromuscular efficiency and sensorimotor modulation [33]. Thus, higher postural balance may be associated with increased perceived physical ability and confidence, thus fostering the development of self-esteem [25,28]. Similar correlations between balance-related perceptions and psychological functioning have been previously recorded in both adolescent and clinical populations [14,43].

Nevertheless, this relationship is likely multifactorial and potentially bidirectional. Adolescents with higher self-esteem, greater confidence, or higher participation in physical activity may also develop better motor competence and postural control over time [12,17,26]. Accordingly, postural balance should not be interpreted as a direct determinant of psychosocial functioning, but rather as one component within a broader network of physical, psychological, and contextual factors influencing adolescent well-being.

The associations observed between postural balance and psychosocial outcomes may also be interpreted within broader motor competence and self-perception frameworks. Previous evidence suggests that greater motor competence is associated with higher perceived physical competence, better self-concept, and improved psychological well-being during adolescence [17,25,26]. In this context, more efficient postural control may contribute to more favorable perceptions of physical capability and functional confidence. These interpretations are also consistent with self-determination theory, which identifies perceived competence as a central contributor to motivation and well-being [13,18,19]. However, the relatively modest R^2^ values observed in the present models indicate that psychosocial outcomes are likely influenced by multiple interacting psychological, social, behavioral, and environmental factors beyond the physical variables included in this study. Therefore, the current findings should be considered preliminary and hypothesis-generating rather than strongly predictive.

Among the secondary exploratory outcomes, greater non-dominant MIHS and younger age were associated with more favorable perceptions of a task-involving motivational climate. Previous studies have shown that task-oriented climates emphasizing effort, cooperation, and personal improvement are associated with more adaptive psychosocial outcomes during adolescence [8,14,19]. The association between greater MIHS and a more favorable motivational climate may reflect underlying mechanisms related to self-confidence, perceived physical competence, and physical self-concept [10,17,25]. However, because this association remains relatively underexplored and the analysis was exploratory in nature, these findings should be interpreted cautiously. In particular, the relationship between non-dominant MIHS and motivational climate should not be interpreted as evidence of a direct functional relationship, but rather as a preliminary associative pattern that warrants confirmation through longitudinal research.

With respect to HRQoL, lower CoP mean velocity under EC conditions, smaller sway area under EO conditions, and male sex were associated with higher HRQoL scores. These findings are consistent with previous evidence suggesting that better physical functioning and motor competence may contribute to more favorable perceptions of well-being in children and adolescents [20,22,24,25,28]. Given that HRQoL is a multidimensional construct encompassing physical, emotional, social, and school-related domains, it is likely influenced by numerous contextual and developmental factors beyond physical fitness alone [22,28]. This interpretation is reinforced by the modest explanatory power of the regression model, suggesting that psychological, familial, social, and environmental variables likely contribute substantially to perceived quality of life during adolescence.

From an applied perspective, the present findings provide preliminary support for the inclusion of motor competence, muscle strength, and postural balance within broader school-based health promotion strategies. Previous evidence has suggested that age-appropriate physical activity environments emphasizing competence, autonomy, and task involvement may positively support adolescent development [14,19,26]. However, these implications should be framed conservatively, as the observed associations do not establish causality. Rather than supporting direct intervention recommendations, the findings suggest that physical fitness and psychosocial functioning may be interconnected during early adolescence. Future longitudinal and intervention-based studies are needed to determine whether improvements in postural balance and motor competence can meaningfully influence psychosocial outcomes over time.

In consideration of all the above, better static postural balance and greater muscle strength were associated with more favorable psychosocial outcomes, particularly self-esteem and HRQoL, in early adolescents. These findings contribute to the growing literature supporting a biopsychosocial perspective of adolescent health by integrating objective physical fitness indicators with psychosocial outcomes in a school-based context [25,26,28]. However, because the observed associations were modest and derived from a cross-sectional design, the findings should be interpreted cautiously as preliminary and exploratory. Future longitudinal and intervention-based studies are necessary to clarify the directionality and underlying mechanisms of these relationships.

### 4.1. Limitations

This study possesses certain limitations that must be acknowledged when analyzing the results. The cross-sectional design precludes causal inferences, necessitating that all revealed associations be considered as associative and exploratory.

Secondly, the pronounced sex disparity (74% male) and the notable age difference between males and females (14.01 ± 1.33 vs. 12.82 ± 1.30 years, *p* < 0.001) may have introduced confounding variables associated with sex, age, and biological maturity. Despite the statistical control of age and sex in all regression models, residual confounding remains a possibility, especially as direct measures of biological maturation, such as Tanner stage or peak height velocity, were not evaluated. The prevalence of male participants may have affected the observed relationships and restricted the generalizability of the findings. Consequently, the findings should not be construed as sex-specific effects, but rather as association patterns identified within a primarily male cohort of early adolescents.

The implementation of a purposeful non-probabilistic sampling method and recruiting from a restricted number of schools limits external validity. Thus, the results must be understood within the specific context of the participating schools in Talca, Chile, and may not be entirely applicable to wider adolescent demographics.

Third, the regression models elucidated only a minor fraction of the variance in psychosocial outcomes (R^2^ = 0.158–0.438), indicating that morphological and physical fitness characteristics contribute only partially to the observed variability. Additional psychological, social, behavioral, and environmental factors certainly influenced the outcomes but were excluded from the current analysis. Despite acceptable multicollinearity diagnostics, significant conceptual overlap among CoP variables may persist, as numerous factors represent interconnected facets of postural regulation. Furthermore, dimensionality-reduction techniques, such as principal component analysis (PCA), were not utilized due to the emphasis on maintaining the biomechanical and clinical interpretability of individual CoP metrics. This should be recognized as a methodological constraint.

The evaluation of static postural balance was conducted utilizing a force platform with a sample frequency of 40 Hz, which is below the rates often advised in current posturography studies. The examined CoP variables predominantly indicate low-frequency postural control; however, the chosen sample frequency may have diminished sensitivity to minor balance variations. Moreover, test–retest reliability indices, including intraclass correlation coefficients, were unavailable, constraining the assessment of measurement stability. All evaluations were performed utilizing defined protocols and calibrated instruments to reduce measurement variability. Future research should include increased sampling frequencies and reliability assessments to enhance methodological rigor.

Ultimately, psychosocial characteristics were evaluated by self-report questionnaires, which may have been affected by social desirability bias, subjective interpretation, and inaccuracies in responses. Validated tools exhibiting satisfactory internal consistency for adolescent groups were employed to enhance measurement reliability.

### 4.2. Strengths

This study possesses numerous significant strengths. Initially, static postural balance was evaluated by objective force platform measurements, enhancing the reliability and accuracy of the functional performance assessment. The study employs a biopsychosocial paradigm by incorporating morphological, physical, functional, and psychological dimensions with validated instruments. The incorporation of static postural balance as a factor related to psychosocial outcomes constitutes a novel contribution that enhances existing knowledge beyond conventional physical fitness evaluations in teenage populations. The amalgamation of objective physical metrics with psychosocial outcomes in a school-based early adolescent cohort offers a significant practical contribution for forthcoming health promotion and physical education research.

## 5. Conclusions

In early adolescence, more favorable postural balance performance and greater muscle strength may be associated with certain psychosocial outcomes, particularly self-esteem and HRQoL. These findings suggest that objective physical fitness indicators, particularly postural balance, may be associated with psychosocial well-being during this developmental stage. In contrast, no significant associations were observed for school climate or ego-involving motivational climate, supporting the importance of broader contextual and social influences on these constructs. The present study contributes to the growing literature adopting a biopsychosocial perspective on early adolescent health by integrating objective physical fitness indicators with psychosocial outcomes in a school-based context. Given the cross-sectional design and exploratory nature of the study, these findings should be interpreted cautiously as exploratory associative patterns and viewed as hypothesis-generating.

## Figures and Tables

**Table 1:** The sample’s morphological variables, maximal isometric handgrip strength and static postural balance characteristics in early adolescents.

Variables		Female (n = 61)	Male (n = 174)	*p* Value	Effect Size	Category
Age (years)		12.82 ± 1.30	14.01 ± 1.33	**<0.001**	0.902	Large effect
Morphological	Body mass (kg)	59.84 ± 2.03	66.58 ± 1.38	**<0.001**	0.909	Large effect
	Bipedal height (m)	1.56 ± 0.08	1.65 ± 0.06	**<0.001**	0.904	Large effect
	BMI (kg/m^2^)	24.35 ± 5.33	24.13 ± 5.20	0.772	0.043	Small effect
	Fat mass (kg)	20.74 ± 6.65	19.33 ± 4.82	0.135	0.263	Small effect
	Fat-free mass (kg)	19.61 ± 3.66	21.50 ± 3.21	**<0.001**	0.567	Moderate effect
MIHS	Dominant (kg)	22.74 ± 4.80	33.19 ± 8.76	**<0.001**	1.314	Large effect
	Non-dominant (kg)	20.83 ± 4.84	31.20 ± 8.47	**<0.001**	1.345	Large effect
Static postural balance	Sway area EO (mm^2^)	0.05 ± 0.03	0.05 ± 0.02	0.831	0.039	Small effect
	Mean velocity EO (m/s)	0.25 ± 0.03	0.26 ± 0.02	0.190	0.242	Small effect
	ML velocity EO (m/s)	0.43 ± 0.15	0.45 ± 0.11	0.400	0.144	Small effect
	AP velocity EO (m/s)	0.62 ± 0.24	0.68 ± 0.20	0.095	0.276	Small effect
	Sway area EC (mm^2^)	0.05 ± 0.07	0.04 ± 0.02	0.449	0.181	Small effect
	Mean velocity EC (m/s)	0.27 ± 0.04	0.28 ± 0.02	0.128	0.324	Small effect
	ML velocity EC (m/s)	0.45 ± 0.12	0.50 ± 0.19	**0.044**	0.303	Small effect
	AP velocity EC (m/s)	0.79 ± 0.28	0.87 ± 0.28	0.067	0.275	Small effect

AP: anteroposterior; BMI: body mass index; EC: eyes-closed; EO: eyes-open; MIHS: maximal isometric handgrip strength; ML: mediolateral; mean velocity: average displacement of CoP per second; ML: mediolateral velocity (x-axis); AP: anteroposterior velocity (y-axis); sway area: 95% confidence ellipse of the CoP trajectory. Note: The age difference between male and female participants is statistically significant (*p* < 0.001) and may reflect differences in school grade distribution. In addition, the sample shows a predominance of male participants (74%), and ages are clustered at the lower end of adolescence (mean age of 13.7 years). Values are presented as mean ± standard deviation (SD). Sex comparisons presented in Table 1 were based on independent-samples *t*-tests and were not adjusted for age. Bold values indicate statistical significance (*p* < 0.05).

**Table 2:** Characteristics of the psychosocial environment in early adolescents.

Variables	Female (n = 61)	Male (n = 174)	*p* Value	Effect Size	Category
Self-esteem	25.033 ± 3.445	26.316 ± 3.366	**0.011**	0.382	Small effect
Task-involving motivational climate	66.689 ± 10.998	59.195 ± 10.780	**0.001**	0.678	Moderate effect
Ego-involving motivational climate	40.656 ± 9.835	46.638 ± 8.668	**0.001**	0.659	Moderate effect
School climate	51.148 ± 5.026	51.201 ± 10.010	0.959	0.005	Small effect
Health-related quality of life	31.689 ± 5.912	35.764 ± 5.574	**0.001**	0.719	Moderate effect

Note: This table presents descriptive statistics of psychosocial variables by sex, serving as the basis for subsequent association analyses. Values are presented as mean ± standard deviation (SD). Bold values indicate statistical significance (*p* < 0.05).

**Table 3:** Multiple linear regression model for self-esteem in early adolescents.

Variables	R^2^	Unstandardized B (95% CI)	Standardized β (95% CI)	*p* Value
Model	0.168			**0.001**
(Constant)		12.165 (5.333–18.998)	—	**0.001**
CoP Sway Area EC (mm^2^)		−35.053 (−56.611–−13.992)	−0.24 (−0.38–−0.10)	**0.001**
CoP mean velocity EC (m/s)		−6.860 (−9.968–−3.752)	−0.27 (−0.40–−0.14)	**0.001**
CoP ML velocity EC (m/s)		−5.138 (−9.598–−0.677)	−0.15 (−0.28–−0.02)	**0.024**

CoP: center of pressure; EC: eyes-closed; ML: mediolateral; CI: 95% confidence interval. Note: This table presents the final multiple linear regression model for self-esteem. Only predictors retained in the final adjusted model are shown. BMI was initially considered but excluded due to severe multicollinearity with body mass and height, as described in the Statistical Analysis Section. Unstandardized (B) and standardized (β) coefficients with 95% confidence intervals (CI) are reported. Sex was dummy coded as 0 = female and 1 = male. Bold values indicate statistical significance (*p* < 0.05).

**Table 4:** Multiple linear regression models obtained for task-involving motivational climate in early adolescents.

Variables	R^2^	Unstandardized B (95% CI)	Standardized β (95% CI)	*p* Value
Model	0.438			**<0.001**
(Constant)		13.182 (11.744–14.620)	—	**<0.001**
Age		−4.485 (−5.503–−3.467)	−0.58 (−0.72–−0.44)	**<0.001**
Nondominant MIHS		−0.181 (−0.349–−0.013)	−0.21 (−0.39–−0.02)	0.034

MIHS: maximal isometric handgrip strength; CI: 95% confidence interval. Note: This table presents multiple linear regression models identifying physical and morphological predictors of task-involving motivational climate. The coefficient B values represent unstandardized regression coefficients. Both unstandardized coefficients (B) and standardized coefficients (β), with their corresponding 95% confidence intervals (95% CI), are reported for each predictor. Sex was dummy coded as 0 = female and 1 = male. Bold values indicate statistical significance (*p* < 0.05).

**Table 5:** Multiple linear regression models obtained for health-related quality of life in early adolescents.

Variables	R^2^	Unstandardized B (95% CI)	Standardized β (95% CI)	*p* Value
Model	0.158			<0.001
(Constant)		18.347 (8.719–27.974)	—	<0.001
CoP Mean Velocity EC (m/s)		−57.581 (−94.289–−20.873)	−0.22 (−0.36–−0.08)	0.002
CoP Sway Area EO (mm^2^)		−30.003 (−47.450–−12.557)	−0.18 (−0.28–−0.07)	<0.001
Sex (Male)		3.592 (1.983–5.200)	0.19 (0.10–0.28)	<0.001

CoP: center of pressure; EC: eyes-closed; EO: eyes-open; CI: 95% confidence interval. Note: This table presents multiple linear regression models identifying physical and morphological predictors of health-related quality of life. Both unstandardized coefficients (B) and standardized coefficients (β), with their corresponding 95% confidence intervals (95% CI), are reported for each predictor. Sex was dummy coded as 0 = female and 1 = male.

## Data Availability

The data presented in this study are available on reasonable request from the corresponding author. The data are not publicly available because they contain sensitive information from minors and are subject to ethical and privacy restrictions established by the institutional ethics committee and informed consent procedures.

## Supplementary Materials

The following supporting information can be downloaded at https://www.mdpi.com/article/10.3390/healthcare14121659/s1, Table S1. Variance inflation factor (VIF) values are predictors in the multiple linear regression model for self-esteem.
