# Supplementary material for: Associations of Physical Fitness and Postural Balance with Psychosocial Well-Being in Early Adolescents: A School-Based Cross-Sectional Study
Source: Healthcare (Basel). 2026 Jun 11;14(12):1659. doi: 10.3390/healthcare14121659 (PMC13300289; doi:10.3390/healthcare14121659)
Supplement: Supplementary file 1 [file healthcare-14-01659-s001.zip › healthcare-4292562-supplementary.pdf]

Supplementary Materials

Table S1. Variance Inflation Factor (VIF) values predictors in the multiple linear regression model for self-esteem.

Predictor Variable	VIF
(Constant)	
Sex	1.64
Age	2.07
Body mass (kg)	2.02
Bipedal height (m)	3.19
Fat mass (kg)	1.60
Fat-free mass (kg)	2.00
MIHS Dominant (kg)	8.40
MIHS Non-dominant (kg)	8.56
Sway Area EO (mm^2)	1.85
Mean velocity EO (m/s)	2.59
ML velocity EO (m/s)	1.77
AP velocity EO (m/s)	1.63
Sway Area EC (mm^2)	2.18
Mean velocity EC (m/s)	3.76
ML velocity EC (m/s)	1.20
AP velocity EC (m/s)	1.90

Note: AP: anteroposterior; CoP: center of pressure; EC: eyes closed; EO: eyes open; MIHS: maximal isometric handgrip strength; ML: mediolateral. The Variance Inflation Factor (VIF) quantifies the severity of multicollinearity. A VIF value below 10 is generally considered acceptable, indicating that multicollinearity does not unduly influence the stability of the regression coefficients.
